# Heart Rate Variability and Autonomic Dysfunction After Stroke: Prognostic Markers for Recovery

**DOI:** 10.3390/biomedicines13071659

**Published:** 2025-07-07

**Authors:** Sara Lago, Toon T. de Beukelaar, Ilaria Casetta, Giorgio Arcara, Dante Mantini

**Affiliations:** 1 Neurophysiology Laboratory, IRCCS San Camillo Hospital, Via Alberoni 70, 30126 Venice, Italy; sara.lago@hsancamillo.it (S.L.); giorgio.arcara@hsancamillo.it (G.A.); 2 Movement Control and Neuroplasticity Research Group, KU Leuven, Tervuursevest 101, 3001 Leuven, Belgium; toon.debeukelaar@kuleuven.be; 3 Neurorehabilitation Department, IRCCS San Camillo Hospital, Via Alberoni 70, 30126 Venice, Italy; ilaria.casetta@hsancamillo.it; 4 Department of General Psychology, University of Padua, Via Venezia 8, 35131 Padua, Italy

**Keywords:** stroke, brain lesion, sensorimotor deficits, cognitive impairments, heart rate variability (HRV), autonomic nervous system (ANS)

## Abstract

Stroke is a major cause of long-term disability and mortality worldwide, often resulting in impairments not only in motor and cognitive functions but also in autonomic nervous system (ANS) regulation. Among the physiological markers that reflect ANS activity, heart rate variability (HRV) has emerged as a promising biomarker for assessing stroke severity and predicting recovery outcomes. HRV quantifies the temporal fluctuations between heartbeats and is traditionally analyzed through time- and frequency-domain measures. More recent approaches have introduced non-linear metrics such as approximate entropy, sample entropy, and detrended fluctuation analysis to capture complex heart rate dynamics. In this narrative review, we address the role of both linear and non-linear HRV parameters in the context of stroke, highlighting their relevance for understanding autonomic dysfunction and guiding rehabilitation. Evidence shows that reduced HRV is associated with poorer functional outcomes, higher mortality, and increased risk of complications post-stroke. Moreover, HRV trends can provide valuable insights into treatment effectiveness and individual recovery trajectories. We also discuss practical considerations for HRV measurement, including device selection, preprocessing strategies, and the need for methodological standardization. Finally, we outline interventional strategies that may enhance HRV and promote better recovery. Together, these findings support the integration of HRV analysis into stroke care as a non-invasive, accessible tool to guide prognosis and tailor interventions.

## 1. Introduction

Stroke is a major cause of disability and death among individuals affected by cerebrovascular disease, representing a critical challenge for both patients and the healthcare system. The impact of stroke extends beyond the immediate medical consequences, affecting the quality of life of patients and placing substantial emotional and financial burdens on their families [1,2]. The rehabilitative process after a stroke is often prolonged and multifaceted, involving a range of therapeutic interventions aimed at improving motor function, cognition, and overall quality of life [3]. Recovery is influenced by numerous factors, including the severity of the initial stroke, the specific brain regions affected, and the patient’s overall health status. Among these factors, autonomic dysfunction is a crucial yet often overlooked element [4,5].

Autonomic dysfunction refers to the impaired regulation of the autonomic nervous system (ANS), which governs involuntary physiological functions such as heart rate, blood pressure, respiration, and digestion. After a stroke, damage to central or peripheral components of the ANS can disrupt the balance between sympathetic and parasympathetic activity [6]. This imbalance may result in cardiovascular instability, arrhythmias, reduced baroreflex sensitivity, and impaired stress adaptation, all of which can adversely affect recovery and increase the risk of complications [4,7]. This dysfunction not only impairs cardiovascular stability but also influences the effectiveness of rehabilitation efforts and overall prognosis. As such, recognizing autonomic dysfunction is essential for optimizing recovery strategies and improving long-term outcomes.

A stroke affects the balance of the main components of the ANS [8]. The sympathetic nervous system (SNS) is responsible for the “fight or flight” response, preparing the body for action by increasing heart rate, dilating pupils, and inhibiting digestion, among other effects. The parasympathetic nervous system (PNS) promotes the “rest and digest” functions, conserving energy by slowing the heart rate, stimulating digestion, and promoting relaxation and recovery. In addition, the enteric nervous system (ENS) controls the digestive system and is sometimes referred to as the “second brain” due to its autonomy and extensive network of neurons. These three components work together to maintain homeostasis, with the sympathetic and parasympathetic systems often having opposing effects to balance bodily functions. An imbalance in the components of the ANS may worsen the patient’s clinical condition [7,9].

A key parameter in evaluating the ANS function is heart rate variability (HRV), which quantifies the time between consecutive heartbeats [10]. HRV offers information about the body’s functional and emotional status [11]. Traditionally, HRV analysis has relied on linear measures from time and frequency domains [12]. Common time-domain measures include the standard deviation of normal-to-normal (NN) intervals (SDNN) and the Root Mean Square of Successive Differences (RMSSD), which denotes the square root of the mean squared differences between successive NN intervals. Frequency-domain analysis, on the other hand, decomposes HRV into its constituent frequencies, offering insights into the contributions of the sympathetic and parasympathetic nervous systems to heart rate regulation. Time- and frequency-domain parameters can be influenced by factors such as health status, stress, and rest, with reduced HRV often indicating autonomic imbalance or diminished physiological resilience. HRV analysis has expanded to include non-linear parameters, providing deeper insights into autonomic regulation [12]. Non-linear parameters, including approximate entropy, sample entropy, and detrended fluctuation analysis (DFA), quantify the complexity, irregularity, and long-range correlations in heart rate dynamics, offering a view of autonomic regulation that complements traditional linear measures. These metrics capture dynamic patterns and interactions in HRV that linear analyses may overlook. By providing a broader perspective on autonomic function, non-linear features may enhance the predictive value of HRV in clinical practice [13].

In this narrative review, we discuss the role of HRV as a potential prognostic biomarker in stroke recovery. By examining both traditional linear measures and advanced non-linear parameters, we highlight how HRV reflects the extent of autonomic dysfunction and its impact on stroke outcomes. Specifically, reduced HRV, observed through time-domain, frequency-domain, and non-linear measures, correlates with poorer recovery, increased mortality, and heightened risk of adverse events related to stroke onset. We explore the mechanisms behind these HRV changes, including neural damage, inflammation, and psychological factors. Additionally, we discuss the clinical utility of HRV for monitoring, risk stratification, and guiding interventions such as pharmacological treatments, rehabilitation, and lifestyle modifications. As HRV analysis evolves, integrating these diverse measures is crucial for optimizing stroke management and improving patient outcomes. Future research should continue to advance HRV techniques and develop targeted interventions to enhance autonomic function and stroke recovery.

## 2. Pathophysiology of Stroke and Autonomic Dysfunction

### 2.1. Types of Stroke: Ischemic and Hemorrhagic

**Figure 1:**
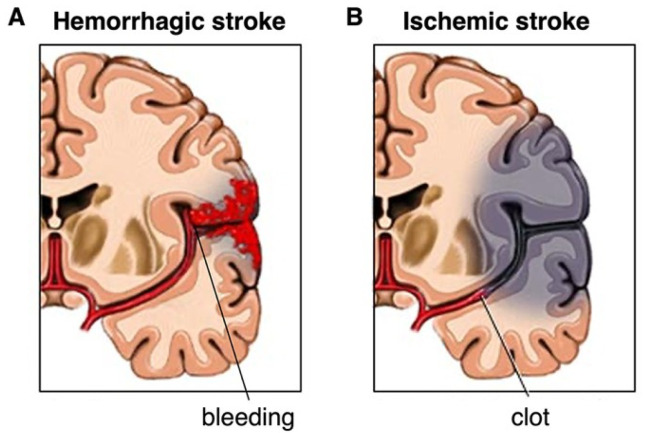
Illustration of hemorrhagic and ischemic strokes. (**A**) A hemorrhagic stroke occurs when a blood vessel bursts, causing bleeding into the brain. (**B**) An ischemic stroke occurs when a blood vessel supplying the brain becomes blocked, as by a clot.

Strokes are broadly classified into two main types, namely, ischemic and hemorrhagic (Figure 1). Ischemic strokes affect about 85% of all stroke patients; they occur when a blood clot blocks an artery in the brain [3]. This obstruction hinders blood flow and oxygen supply, thus causing ischemia-mediated brain tissue damage. The blockage may be thrombotic or embolic in origin. Thrombotic strokes are caused by a clot originating in one of the brain’s arteries, usually due to atherosclerosis. In contrast, in an embolic stroke, the clot is formed somewhere else in the body, such as in the heart, and then travels to the brain.

Hemorrhagic strokes, on the other hand, are caused by the breaking of a vessel in the brain, which results in bleeding in or around the brain [3]. There are two primary types of hemorrhagic stroke: intracerebral hemorrhage and subarachnoid hemorrhage. Intracerebral hemorrhage is a type of bleed that happens when an artery within the brain is ruptured and begins to bleed. Subarachnoid hemorrhage is defined as bleeding that takes place in the subarachnoid space that separates the brain and the fragile membranes enveloping it. Hemorrhagic strokes are secondary to processes like hypertension, aneurysms, arteriovenous malformations, and head injury. Ischemic and hemorrhagic strokes cause severe neurological impairments. As a result, patients develop physical, social, and cognitive disabilities [5].

### 2.2. Impact of Autonomic Dysfunction in Stroke

The ANS plays a critical role in regulating cardiac rhythm, blood pressure, and the characteristics of blood vessels. Following a stroke, ANS dysfunction can occur, manifesting as imbalances between the sympathetic and parasympathetic systems [7,9]. Such imbalances can significantly impact stroke outcomes and may vary between individuals. In stroke patients, changes include altered resting heart rate and blood pressure dynamics, increased variability in these parameters, reduced baroreflex sensitivity, and disrupted autonomic reflexes [9,14]. Baroreflex, which is essential for detecting and responding to blood pressure fluctuations, is particularly affected. Impaired baroreflex function reduces the body’s ability to maintain cardiovascular stability, contributing to greater instability and stress in stroke patients. Consequently, the compromised autonomic reflex arcs lead to a decreased ability to manage neuromuscular stress, further exacerbating cardiovascular instability [15].

Autonomic changes can also predispose the patient to increased mortality, more frequent recurrent strokes, and poorer functional outcomes. Studies have also revealed that post-stroke autonomic dysfunction is directly linked to cardiac arrhythmias, acute myocardial infarction, and sudden cardiac mortality [16]. This underlines the necessity to pay sufficient attention to the assessment of the autonomic function condition in stroke patients to enhance their prognoses. Furthermore, it is important to explore and determine the degree to which autonomic dysfunction occurs in stroke patients depending on the type and severity of the stroke to ease the creation of effective strategies to be employed [5,17]. Through early recognition of autonomic dysfunction in the course of stroke after-effects management, clinicians may enhance the patient’s quality of life and alleviate stroke deficits [3]. Studies revealing the means of restoring the balance of sympathetic and parasympathetic activity, for instance, autonomic modulation therapies, exercise, and stress reduction, may be sought to help boost the quality of life in stroke patients [4,18,19].

## 3. Heart Rate Variability: Measurement and Analysis

### 3.1. Basics of HRV

**Figure 2:**
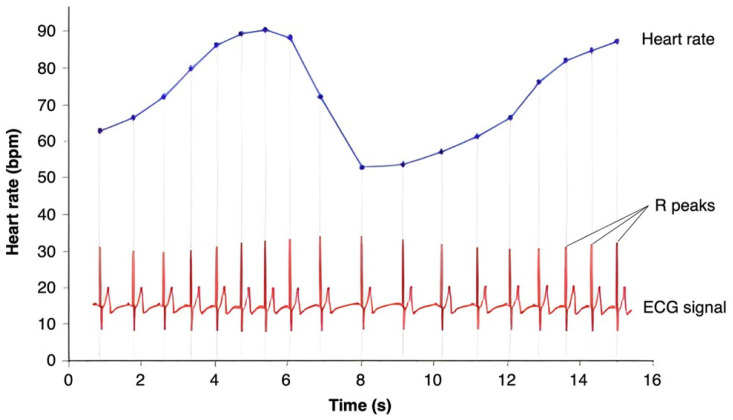
Variations of heart rate over time, derived from the time elapsed between consecutive R peaks. The electrocardiography (ECG) signal is shown on the bottom, whereas the instantaneous heart rate is represented by the blue line. This pattern of heart-rate accelerations and decelerations is the basis of the heart’s functioning.

Heart rate variability (HRV) is a measure of the variation in time between consecutive heartbeats (i.e., RR or inter-beat interval) (Figure 2), reflecting the ability of the heart to adapt to various physiological and environmental factors. Unlike heart rate, which measures beats per minute, HRV captures subtle changes in timing between each beat, providing insights into the ANS’s regulation of the heart. A higher HRV generally indicates a well-functioning, adaptable heart and a balanced nervous system, while a lower HRV can suggest stress, fatigue, or underlying health issues. As such, HRV is often used as an indicator of overall cardiovascular health, stress levels, and physical fitness [20].

### 3.2. Methods of HRV Measurement

**Figure 3:**
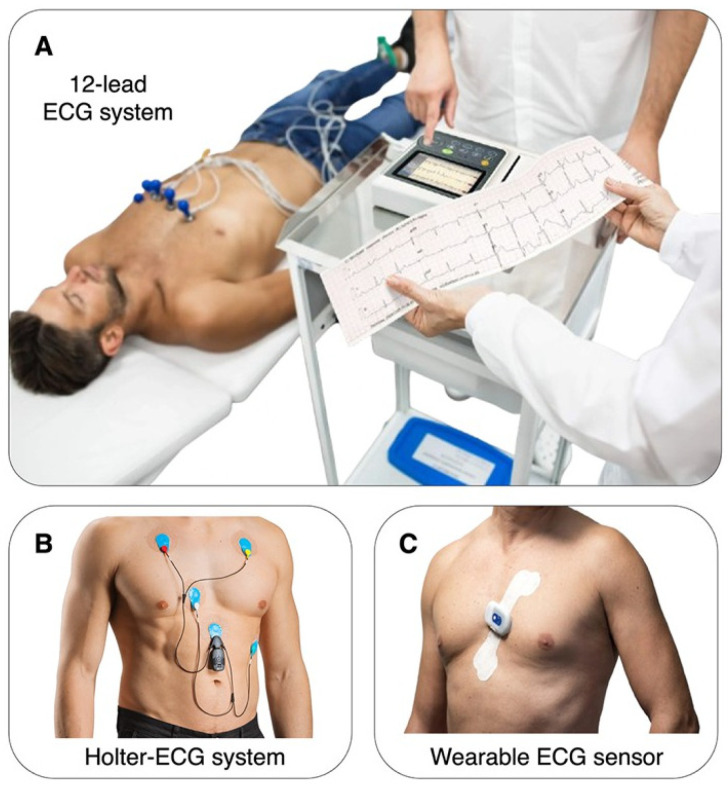
Examples of different systems for ECG measurement. (**A**) A 12-lead ECG system is a medical device to collect short recordings of the heart’s electrical activity from 12 different electrodes placed on the body while the participant is lying. (**B**) A Holter-ECG system is a portable device that uses electrodes attached to the chest and allows for monitoring of the heart’s rhythm and electrical signals during normal daily activities. (**C**) A wearable ECG sensor is a compact device for the monitoring of the heart’s electrical activity during daily activities, which is often embedded in smartwatches, chest straps, or patches.

HRV can be estimated by different devices, each with its peculiarities. The most frequently used devices are standard 12-lead electrocardiograms (ECGs), ambulatory monitoring devices such as Holter monitors, and innovative wearable technologies (Figure 3). Among other parameters, the choice of the device and the measurement duration are the most critical factors affecting the analysis and interpretation of the cardiac signal.

The 12-lead ECG is the most commonly used HRV assessment approach, popularly considered the gold standard. It gives detailed results in a short period and usually takes up to a few minutes. This method is popular in healthcare organizations since it is precise and consistent. The high resolution of ECG makes it easy to identify RR intervals, which are a critical factor in HRV reliability [21]. However, because of the short measurement duration and the need for a controlled clinical environment, it may fail to register long-term variability and the impact of daily activities on heart rate dynamics [22].

Holter monitors are ambulatory tools that capture ECG signals for several hours, commonly twenty-four hours or more. This ability to record for the long term gives a comprehensive evaluation of the HRV and autonomic function both during the day and at night [23]. Holter monitors are mainly preferred because they help detect the rhythms associated with a 24-h day and differing activities, stress, and sleep, all of which interfere with HRV. However, the continuous mode of Holter monitoring can introduce artifacts because of movement and changes in electrode contact with the skin, so data preprocessing is necessary.

Furthermore, there are various modern devices based on the concept of wearable electronics, including smartwatches, fitness trackers, etc., that have transformed the process of monitoring HRV. Such devices offer easy and effective ways of monitoring HRV in the long term in natural conditions [24]. Unlike ECGs, which directly measure the heart’s electrical activity via the QRS complex, most wearables use photoplethysmography (PPG), an optical method that detects blood volume changes to estimate interbeat intervals. PPG-based wearable devices have gained popularity for HRV monitoring due to their convenience and ease of use. These devices estimate HRV by detecting blood volume changes at the skin surface using light sensors. While several studies have demonstrated moderate-to-high correlation with ECG-based HRV under resting conditions, their accuracy can be significantly compromised by movement, irregular light exposure, and sensor placement. Moreover, PPG’s sensitivity to skin tone and ambient lighting presents further limitations for consistent HRV capture [25,26]. Despite these challenges, PPG-based devices are increasingly used for long-term monitoring in real-world settings. Their potential utility in stroke risk stratification lies in enabling passive, continuous data collection in post-discharge patients who may otherwise not undergo regular cardiovascular assessment [27].

### 3.3. HRV Preprocessing

HRV analysis is highly sensitive to artifacts and noise, making it essential to preprocess the cardiac signal before analysis. Artifacts induced by movement or poor electrode contact can introduce irregularities in the ECG signal. Filtering is often used to eliminate high-frequency noise and baseline drift from the ECG signal, which can obscure the true nature of RR intervals [28,29]. Both low-cut and high-cut filtering strategies are employed to define the signal’s band frequency and focus on the time segment of interest, thereby improving the quality of the data. In addition, various algorithms and software tools have been developed to detect and correct these artifacts, which affect the correct detection of heartbeats.

Detection errors, such as false positives (detecting heartbeats that do not exist) or false negatives (missing true heartbeats), can substantially impact the estimation of RR intervals. This issue affects not only ECG but also PPG, leading to inaccurate HRV measures [30]. These detection errors can arise from various sources, including noise, artifacts, or limitations in the detection algorithms. False positives can artificially shorten RR intervals, while false negatives can elongate them, both of which distort the derived HRV metrics. To mitigate these effects, it is crucial to implement correction methods that accurately identify and correct these distortions [31]. One effective approach involves setting realistic assumptions about the duration of individual RR intervals based on preceding intervals, leveraging the inherent regularity of cardiac cycles under physiological conditions. By carefully correcting for these detection errors, the reliability and validity of HRV analysis can be significantly enhanced, providing a more accurate reflection of physiological variations.

### 3.4. Extraction of Traditional Linear HRV Parameters

Traditional HRV analysis encompasses two primary approaches—time-domain and frequency-domain parameters—each offering distinct perspectives on autonomic function and dysfunction. Time-domain parameters provide insights into how much the RR intervals deviate from an average level [12,32]. These parameters are straightforward to compute and understand, making them widely used in both research and clinical practice.

One of the key time-domain parameters is the Mean RR Interval (NN), which represents the average time between consecutive heartbeats. This metric offers a basic view of heart rate over a specified period. Another important parameter is the SDNN, which reflects the total HRV and is influenced by both sympathetic and parasympathetic activities. SDNN calculates the mean of the NN intervals and indicates the variability of the interbeat intervals through their standard deviation. Additionally, the RMSSD is used to assess short-term HRV, primarily reflecting parasympathetic activity. RMSSD is determined as the square root of the average of the squares of the differences between successive NN intervals. Another related parameter is pNN50, which quantifies the proportion of NN intervals that differ by more than 50 milliseconds from the previous interval, serving as another index of parasympathetic tone and short-term HRV [33].

In contrast, frequency-domain parameters describe the power or variance patterns within designated frequency bands of the HRV signal, offering insights into the physiological processes regulating heart rhythm [12,32]. Low-Frequency (LF) power, within the 0.04–0.15 Hz range, mainly represents both sympathetic and parasympathetic activity and is commonly associated with baroreceptor activity and blood pressure regulation. High-Frequency (HF) power, ranging from 0.15 to 0.4 Hz, primarily reflects parasympathetic tone and is linked to respiratory frequency fluctuations in heart rate, making it a key measure of parasympathetic activity. The LF/HF ratio, a widely recognized index, represents the balance between sympathetic and parasympathetic influences. Higher LF/HF values suggest sympathetic dominance, while lower values indicate parasympathetic predominance [34].

### 3.5. Extraction of Non-Linear HRV Parameters

Recent advancements in the field of HRV have introduced novel indices designed to capture the complex, non-linear fluctuations in heart rate dynamics. Among these, Lyapunov Exponent (LE), Approximate Entropy (ApEn), Sample Entropy (SampEn), and Detrended Fluctuation Analysis (DFA) have gained prominence. These non-linear measures provide deeper insights into autonomic regulation that go beyond the capabilities of traditional linear analysis methods [12].

LE measures the sensitivity of the heart rate time series to initial conditions, quantifying how small perturbations can grow over time [35]. A positive LE indicates chaotic behavior, suggesting a highly responsive and adaptable ANS capable of reacting swiftly to environmental changes. This adaptability is crucial for maintaining cardiovascular stability and responding to physiological demands. However, excessively high values may reflect pathological conditions, where heart rate dynamics become overly chaotic and unpredictable. By assessing the degree of chaos and stability in heart rate dynamics, LE offers valuable insights into the balance between order and flexibility in autonomic regulation.

ApEn is a statistical measure that quantifies the complexity and irregularity within a time series by evaluating the predictability of subsequent data points [36,37]. Lower ApEn values indicate more regular and predictable heart rate patterns, often associated with reduced physiological complexity, which is commonly observed in pathological states. Conversely, higher ApEn values reflect greater complexity and variability, suggesting a more adaptable and resilient autonomic regulation. The adaptability of the ANS is crucial for responding to various physiological demands, and ApEn offers a window into this aspect of cardiac function.

SampEn builds upon the foundation laid by ApEn, offering a more refined and robust measure of data complexity. Unlike ApEn, SampEn reduces the influence of data length and sensitivity to noise, making it a more reliable metric for assessing the complexity of HRV signals [38]. SampEn specifically measures the likelihood that similar patterns in a time series will remain consistent as more data points are considered. Higher SampEn values indicate greater complexity and suggest a more sophisticated level of autonomic control, which is essential for maintaining homeostasis in the face of external and internal stressors. SampEn’s improved robustness makes it a preferred choice in clinical and research settings where data quality and consistency vary.

DFA offers a different approach by examining the fractal-like properties and long-range correlations present in HRV data. DFA evaluates the self-similarity of the HRV signal across different time scales, providing insight into the long-term correlations and scaling behaviors of the ANS’s regulation [39,40]. The scaling exponent derived from DFA reflects the degree to which the HRV signal maintains consistent characteristics over multiple scales. Typically, a value around 1.0 suggests healthy autonomic regulation, while significantly lower or higher values may indicate autonomic imbalance or dysfunction. Importantly, DFA is particularly valuable in detecting subtle changes in autonomic function that may not be apparent through traditional linear analysis.

## 4. HRV as a Biomarker in Stroke Recovery

### 4.1. Mechanisms Underlying HRV Changes Post-Stroke

The reasons behind the fluctuations in HRV among stroke patients are both numerous and complex, reflecting the intricate interplay of various physiological processes. Several key factors contribute to these changes in autonomic regulation, including damage to the central nervous system [41,42], inflammatory responses [43,44], and neuroendocrine disruptions [45,46].

When it comes to central nervous system damage, a stroke can have a profound impact on the brain regions responsible for regulating the ANS, particularly the brainstem and hypothalamus [44]. These areas play a crucial role in maintaining the balance between sympathetic and parasympathetic activities, which is essential for stable HRV [47]. When the brainstem is affected—especially areas like the nucleus of the solitary tract and the dorsal motor nucleus of the vagus nerve, both vital for cardiovascular regulation—the result can be a significant disruption in autonomic function. This imbalance between the sympathetic and parasympathetic nervous systems often leads to a noticeable decrease in HRV.

Another major factor influencing HRV variability post-stroke is systemic inflammation. Stroke triggers an inflammatory response that does not just stay local but can rather affect the ANS as well [48]. Pro-inflammatory cytokines such as interleukin-6 (IL-6) and tumor necrosis factor-alpha (TNF-α) can impair parasympathetic signaling and enhance sympathetic output by acting on brain regions that regulate autonomic balance, such as the hypothalamus and brainstem. These effects contribute directly to the observed reductions in HRV after stroke, beyond mere association [49,50]. This connection becomes even more significant when considering that post-stroke chronic inflammation can prolong autonomic disturbances, further lowering HRV and complicating the recovery process. In other words, the inflammation that follows a stroke is not just a byproduct. It actively hampers the autonomic system’s ability to regain balance, which in turn affects the patient’s overall prognosis.

Neuroendocrine disturbances also play a critical role in altering HRV after a stroke. The disruption of the hypothalamic–pituitary–adrenal (HPA) axis and the production of stress hormones like cortisol and norepinephrine are particularly impactful [45,51]. These hormones are part of the body’s natural stress response, but when they are overproduced, as often happens after a stroke, they can negatively impact autonomic regulation. The chronic stress that follows a stroke, driven by these elevated hormone levels, is strongly linked to decreased HRV. This ongoing imbalance not only reflects the body’s struggle to cope with the stress but also poses a significant challenge to the rehabilitation process [52]. The HPA axis, which is central to regulating stress and maintaining homeostasis, becomes a critical factor here; its dysfunction can affect almost every organ and system in the body, further complicating the patient’s recovery.

### 4.2. Utility of HRV as a Prognostic Tool in Stroke

HRV has emerged as a promising non-invasive biomarker for predicting outcomes in stroke patients. Lower HRV has been consistently associated with increased mortality, greater stroke severity, and reduced potential for recovery. For example, specific HRV measures such as SDNN and RMSSD are significantly lower in patients who do not survive the first 30 days following a stroke [42,53]. Additionally, autonomic function assessed through HRV during the early post-stroke period (1–3 and 7–10 days) has been independently linked to poor three-month outcomes in patients undergoing intravenous thrombolysis [54].

Beyond mortality, HRV appears closely related to stroke severity. Patients with reduced HRV often exhibit larger infarct volumes and more pronounced motor deficits [55]. In cases of stroke-in-evolution (SIE), complexity index values of HRV are significantly lower, whereas higher values are associated with reduced risk of SIE, indicating the potential of HRV as an early warning marker for worsening neurological status [55].

HRV is also a potential indicator of functional recovery. Patients with diminished HRV tend to show poorer responses to motor rehabilitation [56]. Conversely, strategies aimed at improving autonomic function, such as vagus nerve stimulation, are linked to enhanced rehabilitation outcomes [57]. These findings suggest that HRV could play a valuable role not only in acute risk stratification but also in guiding longer-term recovery planning.

## 5. Clinical Applications

HRV can serve as a diagnostic tool for assessing stroke prognosis, particularly because ANS dysfunction plays a crucial role in determining patient outcomes [58]. Understanding the mechanisms behind HRV changes and integrating HRV measurements into routine clinical practice could enhance stroke care. HRV can guide the customization of treatment plans and management strategies, ultimately leading to improved outcomes in stroke patients.

### 5.1. Risk Stratification Based on HRV

Research supports HRV as an effective tool for stratifying stroke patients based on their risk levels. Decreased HRV predicts post-stroke complications, including additional cardiovascular issues or recurrent strokes [59]. This stratification is particularly crucial in the first few days following a stroke, when timely intervention can significantly influence outcomes.

Low HRV levels typically indicate impaired ANS regulation and are associated with a higher mortality rate. Patients with reduced HRV may need closer monitoring and targeted interventions to prevent complications, as they are in a higher-risk category [24]. For example, using HRV in acute stroke cases may help predict the development of complications such as arrhythmias or cardiovascular dysfunction [54]. Conversely, relatively higher HRV values are generally associated with better autonomic regulation and a lower risk of severe complications.

It is important to consider the substantial inter-individual and intra-individual variability in HRV, both of which impact its clinical interpretation. Inter-individual variability arises from stable characteristics such as age, sex, fitness level, baseline autonomic tone, and psychological traits (e.g., trait anxiety and emotion regulation capacities), all of which may influence resting HRV values even in the absence of disease [60,61]. In contrast, intra-individual variability reflects short-term fluctuations in HRV due to circadian rhythms, posture, physical activity, sleep, and emotional state [62,63]. These temporal changes may obscure pathological signals unless longitudinal or standardized measurements are applied. In the context of stroke, the absence of individual pre-stroke HRV baselines complicates interpretation. Thus, personalized baseline assessments, where feasible, and within-subject tracking over time are critical to distinguishing clinically meaningful changes from natural variability. Such contextualization supports the utility of HRV as a reliable prognostic biomarker within a biopsychosocial framework.

### 5.2. Monitoring Recovery Using HRV

Heart rate variability (HRV) is an increasingly valuable biomarker for assessing the recovery process in stroke patients. By tracking fluctuations in HRV characteristics over time, clinicians can gain insights into how well patients are responding to various therapeutic interventions. HRV reflects the dynamic balance between sympathetic and parasympathetic nervous system activity, making it particularly relevant for monitoring post-stroke autonomic function, a key factor in overall recovery.

**Figure 4:**
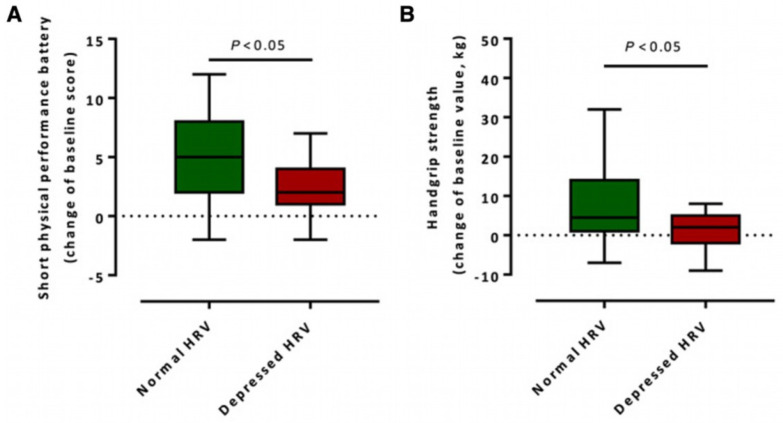
Difference in functional outcome after rehabilitation in patients with normal HRV and depressed HRV. The figure shows the change from baseline value after motor rehabilitation in the (**A**) short physical performance battery and (**B**) handgrip strength. The figure is adapted from [56], with permission.

Improvements in HRV parameters, such as increased high-frequency power or normalized time-domain indices, may signal the gradual restoration of autonomic balance and better physiological resilience. For example, stroke patients with preserved or improved HRV after motor rehabilitation were found to have better functional outcomes in the short physical performance battery and handgrip strength tests compared to those with reduced HRV (Figure 4). This suggests that HRV may serve not only as a passive indicator but also as a responsive metric tied to therapeutic success [56].

Growing evidence supports the use of HRV as a feedback tool to evaluate the effectiveness of rehabilitation programs. Regular HRV assessments enable clinicians to individualize rehabilitation protocols to maximize functional gains and recovery speed [56,57]. For instance, if a patient’s HRV stagnates or declines during therapy, it may indicate insufficient autonomic recovery or overexertion, prompting clinicians to adjust the regimen by incorporating more cardiovascular conditioning, stress management techniques, or adjusting intensity to match physiological capacity.

Moreover, HRV-guided treatment adjustments enhance the responsiveness and adaptability of rehabilitation programs. Continuous HRV monitoring can flag acute changes in autonomic function, allowing for early intervention and preemptive changes in therapy plans before clinical deterioration becomes apparent [13]. This dynamic, patient-centered approach facilitates precision rehabilitation, aligning treatment with each patient’s evolving needs and contributing to better long-term outcomes.

### 5.3. Interventional Strategies to Improve HRV

Numerous interventional approaches have been suggested to augment HRV and facilitate stroke recovery, encompassing the regulation of sympathetic/parasympathetic balance. For instance, several drugs have demonstrated well-established or promising effects to modulate autonomic tone, including beta-blockers and acetylcholinesterase inhibitors. Beta-blockers are known to reduce sympathetic tone while enhancing parasympathetic tone, which positively influences HRV [64]. Similarly, cholinergic-modulating drugs, particularly cholinesterase inhibitors, have been explored for their ability to enhance vagal tone, thereby improving HRV [65]. These pharmacological interventions can be integrated into multidisciplinary stroke management strategies to enhance ANS regulation and promote recovery across various types of stroke.

Behavioral therapies such as exercise and mindfulness training have also proven effective in enhancing HRV and improving clinical outcomes in stroke survivors. While the intensity and timing of physical exercise must be carefully adapted to individual stroke severity and recovery stage, it can support improvements in autonomic function and, over time, cardiovascular fitness. Research shows that regular physical activity, when feasible, enhances parasympathetic activity and modulates sympathetic activity, thereby improving autonomic tone [66,67]. Aerobic activities such as walking, cycling, and swimming can help rebalance the autonomic nervous system by increasing parasympathetic tone and reducing sympathetic dominance over time. However, the acute effects of these exercises on HRV depend on the intensity and duration of the activity. For example, a gentle walk in nature may acutely increase HRV, while prolonged or high-intensity exercise, such as a 3-h bike ride, can temporarily suppress HRV due to increased physiological stress [68]. Strength training and flexibility exercises also contribute to improved autonomic function and may positively impact HRV when appropriately tailored to the individual’s capacity. Notably, acute resistance exercise has been shown to reduce parasympathetic modulation temporarily, especially at higher intensities [69]. In clinical populations, lower-intensity resistance exercise appears to produce more favorable effects on HRV than high-intensity resistance training [70].

Mindfulness practices, including meditation, deep breathing, yoga, and tai chi, can improve HRV by reducing psychological stress and promoting relaxation [71]. These practices are particularly beneficial for addressing the psychological challenges associated with stroke, leading to improved autonomic function and quality of life. For instance, mindfulness-based interventions have shown promise in stroke rehabilitation, with preliminary evidence suggesting potential improvements in HRV, reductions in anxiety and depression, and enhanced quality of life. However, findings remain tentative due to the limited number and methodological variability of existing studies [72].

## 6. Future Directions

Future research should aim to consolidate HRV’s role in stroke rehabilitation by addressing three areas: longitudinal monitoring, mechanistic understanding, and multimodal integration. These efforts may strengthen HRV’s clinical utility and improve prognostic accuracy.

### 6.1. Longitudinal Studies

Longitudinal studies are essential for understanding the long-term impact of HRV on stroke recovery outcomes [66]. These studies should focus on assessing improvements in key recovery areas, such as motor function, cognition, and quality of life in stroke patients. Since most research on HRV variations spans periods of weeks or months, it is crucial to identify patterns and predictors in the recovery process, which can then be applied in clinical practice [73]. Longitudinal data, in particular, offer valuable insights into the temporal aspects of autonomic regulation and how it interacts with different phases of stroke rehabilitation.

The strength of longitudinal studies lies in their ability to provide detailed information on the changes in HRV throughout a patient’s recovery. For instance, these studies can track the temporal dynamics of HRV from the acute stage of stroke through to the chronic stage, identifying factors that influence these changes [74]. Additionally, such research can evaluate the effects of various interventional strategies on HRV and, by extension, on recovery outcomes [54,57]. Longitudinal HRV monitoring may guide personalized interventions in stroke care.

### 6.2. Mechanistic Studies

Further biochemical and cellular research is needed to elucidate the physiological factors that drive changes in HRV among stroke patients. Understanding these mechanisms will help identify potential treatment targets and improve the effectiveness of therapeutic strategies. For example, studying the relationship between inflammatory processes in stroke and shifts in HRV could reveal insights into neuroendocrine and central nervous system damage, as well as other aspects of autonomic dysfunction [75,76]. By gaining a deeper understanding of ANS regulation and its imbalances, clinicians can develop more precise therapies to modulate the ANS and enhance the recovery process.

Research into the neural substrates underlying HRV changes can also provide clinicians with valuable insights into the impact of stroke on the ANS. By combining neuroimaging and electrophysiological studies, researchers can develop a comprehensive understanding of the mechanisms involved in autonomic dysfunction post-stroke. Functional magnetic resonance imaging can be used to observe brain activity related to HRV variations, identifying specific regions involved in autonomic regulation following stroke [77,78]. Conversely, diffusion magnetic resonance imaging can provide insights into white matter integrity and connectivity between autonomic centers in the brain [79]. By studying these connections, researchers can identify disruptions caused by stroke and their impact on HRV, pinpointing crucial neural pathways for autonomic function. Furthermore, the relationship between neural activity and HRV changes can be analyzed using electroencephalography to examine brain wave patterns. This approach helps identify specific neural oscillations associated with autonomic dysfunction, offering a deeper understanding of the neural mechanisms underlying HRV changes [80,81].

### 6.3. Integration with Complementary Biomarkers

Future studies should prioritize combining HRV with other biomarkers and clinical indicators to enhance the specificity of prognoses and optimize patient management. While HRV has shown potential as a non-invasive marker of autonomic function, its predictive power can be substantially enhanced when combined with other physiologic and biochemical indicators. Stroke is a systemic disorder with multisystem involvement—including inflammation, cardiac stress, and neurovascular injury—and a multimodal approach to prognostication is more likely to capture this complexity.

Inflammatory biomarkers, particularly C-reactive protein (CRP) and IL-6, have been independently associated with poor stroke outcomes and reduced HRV. These cytokines are key mediators in the acute phase response and may contribute to impaired parasympathetic tone and sustained sympathetic activation. Elevated CRP and IL-6 levels not only correlate with infarct volume and mortality but also mirror autonomic dysregulation, suggesting a pathophysiological link between inflammation and HRV suppression [82].

Cardiac biomarkers, such as troponin and brain natriuretic peptide (BNP), are also clinically relevant. Troponin elevations are observed in up to 20–30% of acute ischemic stroke patients, even in the absence of overt coronary disease, often reflecting neurogenic myocardial injury or concurrent cardiac dysfunction. Similarly, BNP is linked to atrial fibrillation, cardioembolic stroke subtype, and poor long-term outcomes [83]. When considered alongside HRV data, these markers can help distinguish between centrally mediated autonomic dysfunction and primary cardiac pathology, which may influence both acute management and rehabilitation planning [41].

Neuroimaging markers provide anatomical and structural insights that complement physiological measurements. For instance, strokes affecting the insula, hypothalamus, or brainstem are more likely to disrupt autonomic regulation and produce lower HRV. Infarct volume and lesion location based on magnetic resonance imaging (MRI) can therefore contextualize HRV changes and improve patient stratification when combined in predictive models [20]. A growing number of studies support this integrative approach. For example, a composite risk model incorporating HRV, CRP, and BNP was proposed, which more accurately predicted complications such as post-stroke infections and delayed neurological deterioration [84]. Similarly, it was found that HRV alone underestimates risk in complex patients and that combining it with systemic and neuroimaging data significantly improves prognostic accuracy [85].

Beyond biological markers, advanced data integration techniques, including machine learning and multimodal data fusion, offer a promising path forward. By combining HRV with clinical scores of stroke severity, lab values, and imaging metrics, these systems can personalize prognostication and rehabilitation pathways. In the future, wearable devices and point-of-care diagnostics may further facilitate real-time integration of HRV with biochemical markers, enhancing early detection of complications and enabling dynamic risk assessment.

### 6.4. Methodological Challenges

Despite its clinical and research potential, HRV measurement is subject to several methodological challenges that can impact its interpretation and utility, especially in stroke rehabilitation contexts. HRV is highly sensitive to numerous external and physiological factors, including nutrition and hydration status, caffeine and alcohol intake, physical activity, time of day, posture, respiration rate, and sleep quality [86]. These variables introduce substantial within- and between-subject variability, making it complex to distinguish pathological changes from normal physiological fluctuations, particularly in patients without pre-stroke HRV baselines.

Hence, standardization of measurement protocols is essential to minimize confounding factors and improve data comparability across studies. For example, resting-state HRV should ideally be recorded at the same time of day, under consistent environmental conditions, and following strict pre-assessment guidelines (e.g., fasting, no recent exercise, and controlled breathing) to enhance reliability [12].

Additionally, different HRV metrics (time-domain, frequency-domain, and non-linear) are influenced by varying physiological processes and measurement durations, adding complexity to interpretation. Short-term (e.g., 5-min) and ultra-short-term (e.g., <1-min) HRV recordings, often used in wearable technology and clinical settings, may lack the robustness of 24-h recordings for certain indices [87]. Inconsistent use of these metrics across studies further hampers generalization and clinical translation.

However, recent research has demonstrated that, when properly standardized, HRV measurements can exhibit strong reliability [88]. For instance, a recent study using wearable technology showed high intra-individual consistency of HRV during sleep and resting states, supporting its value for longitudinal monitoring [89]. This progress underscores the importance of rigorous methodology and careful contextual interpretation in both clinical and research applications of HRV. To advance HRV as a reliable biomarker in stroke care, future studies must prioritize methodological transparency, standardized protocols, and reporting guidelines. Doing so will help ensure more accurate, reproducible results and facilitate clinical adoption.

## 7. Conclusions

HRV is recognized as a key prognostic factor in stroke recovery, as it provides insight into autonomic modulation and its critical influence on cardiovascular stability, neurological function, and overall health status. By utilizing both conventional time-domain and frequency-domain parameters, along with novel non-linear methods, clinicians can gain a comprehensive understanding of autonomic activity. These aspects can be combined to provide precise measures that reflect the dependence of autonomic dysfunction on stroke severity, mortality rate, and functional recovery. There is evidence from human clinical trials proving that HRV is helpful in stroke patient management and risk differentiation. HRV could play a pivotal role in clinical practice by guiding therapeutic interventions to improve patient outcomes. This is encouraging because as more healthcare practitioners integrate HRV into their practice, they can develop rehabilitation and stroke therapies that are better aligned with the patient’s recovery needs, ultimately enhancing overall healthcare delivery.

The next step involves conducting further research that refines the current methodology and deepens the theoretical understanding of HRV in stroke. Future studies should explore the interactions between HRV and other biomarkers, such as neuroimaging data, genetic factors, and inflammatory markers, to gain a more comprehensive understanding of stroke pathology and recovery. Additionally, longitudinal studies are essential to determine the long-term impact of HRV on stroke outcomes, which could lead to more effective prognostic models and treatment strategies. The ability to collect HRV data through wearable sensors, such as smartwatches or fitness bands, enhances its practicality as a health marker, offering an accessible, cost-effective, and potentially high-impact means of continuous monitoring.

In conclusion, the use of HRV as a predictive tool enables stakeholders in stroke care to improve the nature of existing management in this specialty. It also helps to advance the quality of treatment and care of stroke patients and enhances the quality of their lives. Since more knowledge, experience, and research about HRV continue to grow, HRV analyses will become a part of modern healthcare practices for stroke, with the potential to significantly improve patient outcomes.

## Abbreviations

The following abbreviations are used in this manuscript:

ANS	Autonomic Nervous System
ApEn	Approximate Entropy
BNP	Brain Natriuretic Peptide
CRP	C-Reactive Protein
DFA	Detrended Fluctuation Analysis
ECG	Electrocardiogram
ENS	Enteric Nervous System
HF	High Frequency
HPA Axis	Hypothalamic–Pituitary–Adrenal Axis
HRV	Heart Rate Variability
IL-6	Interleukin 6
LE	Lyapunov Exponent
LF	Low Frequency
LF/HF	Low Frequency/High Frequency Ratio
MRI	Magnetic Resonance Imaging
pNN50	Proportion of NN Intervals that Differ by More Than 50 ms
PNS	Parasympathetic Nervous System
PPG	Photoplethysmography
RMSSD	Root Mean Square of Successive Differences
SampEn	Sample Entropy
SDNN	Standard Deviation of Normal-to-Normal Intervals
SIE	Stroke-in-Evolution
SNS	Sympathetic Nervous System
TNF-α	Tumor Necrosis Factor-alpha
